# Burden of sequelae and healthcare resource utilization in the first year of life in infants born with congenital cytomegalovirus (cCMV) infection in Germany: A retrospective statutory health insurance claims database analysis

**DOI:** 10.1371/journal.pone.0293869

**Published:** 2023-11-16

**Authors:** Marion de Lepper, Anna-Janina Stephan, Regine Wölle, Wei Wang, Christian Jacob, Kim Maren Schneider, Horst Buxmann, Rangmar Goelz, Klaus Hamprecht, Peter Kummer, Susanne Modrow, Wolfgang Greiner, Agnes Luzak, Miriam Reuschenbach

**Affiliations:** 1 Department of Medical Affairs, MSD Sharp & Dohme GmbH, Munich, Germany; 2 Department of Market Access, MSD Sharp & Dohme GmbH, Munich, Germany; 3 Center for Observational and Real-World Evidence (CORE), Merck & Co., Inc., Rahway, NJ, United States of America; 4 EU Real World Evidence, Xcenda GmbH, Hannover, Germany; 5 Division for Neonatology, Department for Children and Adolescents, Main-Kinzig-Kliniken GmbH, Gelnhausen, Germany; 6 Department of Neonatology, University Children´s Hospital Tuebingen, Tuebingen, Germany; 7 Institute for Medical Virology and Epidemiology of Viral Diseases, University of Tuebingen, Tuebingen, Germany; 8 Department of Otolaryngology, Section of Phoniatrics and Pediatric Audiology, University Hospital Regensburg, Regensburg, Germany; 9 Institute of Medical Microbiology and Hygiene, University Hospital Regensburg, Regensburg, Germany; 10 Department of Health Economics and Health Care Management, Bielefeld School of Public Health, Bielefeld University, Bielefeld, Germany; 11 Global Medical and Scientific Affairs, MSD Sharp & Dohme GmbH, Munich, Germany; Bangabandhu Sheikh Mujib Medical University (BSMMU), BANGLADESH

## Abstract

**Background:**

Congenital cytomegalovirus (cCMV) infection can have a broad range of manifestations. This study aimed to assess cCMV-associated sequelae and healthcare resource utilization (HCRU) in infants during the first year of life in Germany.

**Methods:**

A retrospective, controlled cohort study using German claims data from the Institute for Applied Health Research Berlin (InGef) database was conducted. cCMV-associated sequelae and HCRU during the first year of life were assessed by matching (1:60) infants with at least one inpatient/outpatient cCMV diagnosis (ICD-10-GM: P35.1) ≤90 days after birth (cCMV_90_ cohort) and infants with at least one inpatient cCMV diagnosis plus specific sequelae ≤21 days after birth (cCMV_21-S_) to infants without cCMV or CMV (ICD-10-GM: B25) diagnosis (control group), respectively. Outcomes were analyzed during the first 365 days of life.

**Results:**

Between 2014–2018, we identified 54 newborns for cCMV_90_ and 24 newborns for cCMV_21-S_ cohort. Compared to the 3,240 and 1,440 controls, respectively, more cCMV_90_ infants (83.3% vs. 41.9%, p<0.01) presented with at least one sequela during the first year of life, including intrauterine growth retardation (42.6% vs. 5.3%, p<0.01), sensorineural hearing loss (SNHL) to deafness (38.9% vs. 2.2%, p<0.01), and motor development disorders (33.3% vs. 10.9%, p<0.01). Further, 13.0% of cCMV_90_ infants (vs. 2.3%, p<0.01) suffered from visual impairment. In cCMV_21-S_ cohort, intrauterine growth retardation (79.2% vs. 6.0%, p<0.01), prematurity (54.2% vs. 7.3%, p<0.01), and motor development disorders (50.0% vs. 11.0%, p<0.01) were the most frequent sequelae. Infants in the cCMV_90_ and cCMV_21-S_ cohort had, on average, 7.3 times and 9.5 times more hospitalizations and 2.0 times and 2.1 times more outpatient physician visits than their respective controls (p<0.01). Hospitalized infants with cCMV stayed, on average, significantly longer in hospital compared to their controls (cCMV_90_ cohort: 30.3 days vs. 9.0 days, p<0.01; cCMV_21-S_ cohort: 46.5 days vs. 9.3 days, p<0.01).

**Conclusions:**

cCMV-infection shows a considerable disease and healthcare burden during the first year of life. More than 80% of the identified newborns with cCMV suffered from at least one associated sequela during the first year of life, including long-term sequelae such as SNHL (40%) and visual impairment (13%). Additional steps for prevention of cCMV infection and associated sequelae as well as a comprehensive monitoring of disease burden are needed.

## Introduction

Human cytomegalovirus (CMV) is a double stranded, enveloped DNA virus that belongs to the Herpesviridae family [1]. Worldwide, CMV is estimated to have a seroprevalence of 83% in the general population [2]. In Germany, the overall CMV seroprevalence in the adult population is found to be 56.7% [3]. A recent meta-analysis estimated that globally 0.67% of individuals are congenitally infected with CMV [4]. In Germany, estimates suggest that 0.2–0.5% of all living newborns are congenitally infected [5].

It is important to distinguish between postnatal and congenital CMV (cCMV) infection, as the long-term complications and treatment options differ considerably [6]. While postnatal CMV infection has been considered to be of little clinical consequence in term infants [7], cCMV infection, on the other hand, can cause childhood disabilities, including neurological damage, growth retardation, sensorineural hearing loss, visual impairment and microcephaly [8–11]. Compared to the incidence of other well-known congenital disabilities such as Down syndrome, neural tube defect, or fetal alcohol syndrome, cCMV appears to be more common, but public awareness seems to be lower [1, 12–14].

Approximately 12–20% of congenitally infected newborns are symptomatic at birth, with mortality rates <5% [11, 15, 16]. The percentage of symptomatic children with long-term sequelae is estimated at 40-58% [11].

Only limited real-world data exist on the disease burden of cCMV sequelae and the associated healthcare burden in infants with cCMV in Germany. Rütten et al. described up to 0.04% clinically relevant cCMV infections in all births in a retrospective study in Saxony-Anhalt, Germany [13]. However, nationwide data is still lacking.

The present work covers one part of a large retrospective study that assessed the burden of cCMV in terms of sequelae, healthcare resource utilization (HCRU) and healthcare costs in infants during the first and second year of life from the perspective of the German statutory health insurance (SHI) [17]. In this paper, we present the analysis of cCMV-associated sequelae and HCRU in infants with cCMV diagnoses compared to infants without cCMV in the first year of life (1–365 days). Additional data for cCMV-associated sequelae and HCRU in the second year of life (366–730 days) can be found in the see S1–S5 Tables.

## Materials and methods

### Study design

Using a retrospective, controlled matched cohort design, we analyzed German SHI data from 2014–2019. Burden of cCMV was assessed in terms of cCMV-associated sequelae and HCRU in infants during their first year of life (1–365 days).

### Database

In Germany, health insurance is mandatory, and approximately 88% of the German population, corresponding to about 73 million individuals, is insured under one of 110 public SHIs (as of 2018) [18]. Since 1996, all individuals insured by the SHI are free to choose their health insurance fund. All public SHIs offer the same comprehensive benefits package, as the contents to be reimbursed are defined in Social Law.

We utilized the Institute for Applied Health Research Berlin (InGef) database with claims data from about 60 different SHIs covering approximately 8 million lives insured in one of the contributing SHIs (mainly company or guild health insurances). The InGef database has a well-distributed geographic representation of the German population and good external validity in terms of morbidity, mortality, and drug use [19, 20]. Newborns identified in the InGef database (females 48.6%, males 51.4%) represented 7.8% (n = 298,112) of live births in Germany between 2014-2018 with a similar sex distribution as the German population (females 48.7%, males 51.3%) [21]. The database includes information on all different healthcare sectors such as the inpatient, outpatient, and the pharmacy sector. Data generation occurs routinely during health service provision or during the reimbursement process and reflects the actual utilization of health services from the payers’ perspective across various providers and sectors.

### Ethics approval and consent to participate

Claims data from the participating SHIs are joined in a specialized trust center, anonymized, and transferred to InGef before the data are made available for research. In accordance with the “GPS–Good Practice in Secondary Data Analysis” (Guideline 1: Ethics) [22], the analysis of claims data from the SHI is permitted and does not require the approval of an ethics committee.

### Study population

All newborns available in the InGef database from 2014–2018 were considered for this study. Newborns needed to be continuously observable for at least 365 days of life, except for infants who deceased. Infants with pre-defined diagnoses (leukemia, human immunodeficiency virus (HIV), solid organ transplant, or stem cell transplant) during the first 365 days of life were excluded from the study population (see S6 Table).

Two cCMV cohorts and one control group were defined:

### ■ Study cohorts

- cCMV-cohort 1 (cCMV_90_): all infants with a documented ICD-10-GM (International Classification of Diseases, 10^th^ Revision, German Modification) diagnosis for cCMV (P35.1) during the first 90 days of life irrespective of documented clinical symptoms
- cCMV-cohort 2 (cCMV_21-S_): all infants from cCMV cohort 1 with a documented inpatient ICD-10-GM diagnosis for cCMV (P35.1) and at least one cCMV-specific symptom recorded during any hospital admission in the first 21 days of life (including birth)

### ■ Control group

- all infants without a documented ICD-10-GM diagnosis for cCMV (P35.1) and CMV (B25) at any time during their individual observation period in the database

A more detailed description of selection criteria including information on the German claims data coding system can be found in the S1 File.

A direct 1:60 matching without replacement based on gender, year-specific quarter of birth, and observability of the newborns was performed. Cases needed to be continuously observable for at least 365 days of life except for infants who died during the follow-up. If cases deceased during follow-up, these infants were analyzed until death. Since controls needed to be continuously observable for at least as long as their matched cCMV cases, controls were censored at date of death of the matched case. No controls that deceased during the follow-up period were selected for the matching. No cases were lost during the matching procedure. The matching and data analysis were conducted using “R 3.5.0” software.

### Outcomes

Pre-defined sequelae associated with cCMV (based on clinical expertise and published literature [23]) were identified by ICD-10-GM codes in the outpatient (verified diagnoses) and inpatient (primary or secondary diagnoses) setting (see S7 Table) and reported as the number and percentage of infants with at least one respective comorbid diagnosis related to cCMV observed during the first year of life.

HCRU was analyzed regarding inpatient and outpatient care. For inpatient care we analyzed proportions of infants with hospitalizations, frequency of hospitalizations (in all matched infants), length of stay (in matched infants with hospitalizations), and reasons for hospitalizations defined as primary inpatient diagnosis (3-digit ICD-10-GM code).

For outpatient care we analyzed frequency of physician visits, proportions of infants with and frequency of physical and occupational therapy, and proportions of infants with outpatient pharmaceutical prescriptions identified by 7-digit Anatomical Therapeutic Chemical (ATC) classification codes. Reasons for outpatient visits were summarized based on verified 3-digit ICD-10-GM codes.

### Statistical analysis

Descriptive analyses were performed for patient demographics, cCMV-associated sequelae, and HCRU in terms of mean values, medians, ranges, and standard deviations of continuous variables of interest and frequency distributions for categorical variables. For continuous variables, mean differences with respective 95% confidence intervals were additionally calculated.

Statistical significance of observed numerical differences in outcomes between cohorts was assessed using Mantel–Haenszel matched-pairs test for dichotomous variables and Wilcoxon rank-sum test for continuous variables. P-value <0.05 was considered as statistically significant.

## Results

### Study population

**Fig 1:**
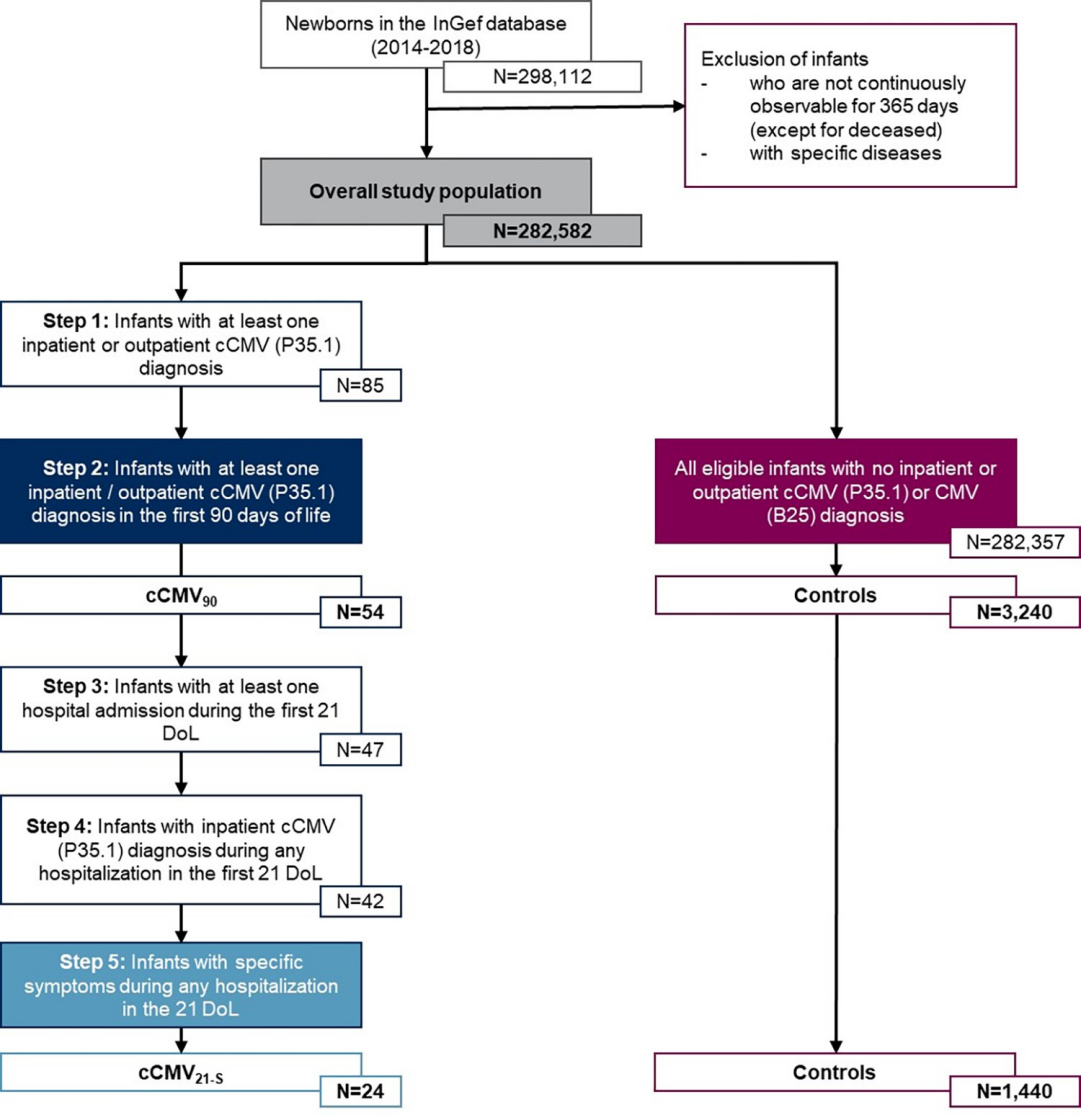
Patient selection process–First year of life (1–365 days). InGef, Institute for Applied Health Research Berlin; cCMV, congenital cytomegalovirus; cCMV_90_, infants with cCMV diagnosis during the first 90 days of life; cCMV_21-S_, infants with inpatient cCMV diagnosis and symptoms during the first 21 days of life; Controls, infants with no cCMV or CMV diagnosis in the observation period; DoL, days of life.

We identified 54 and 24 infants born 2014–2018 for the cCMV_90_ and cCMV_21-S_ cohorts, respectively (Fig 1). 25.9% and 50.0% of newborns in the cCMV_90_ and the cCMV_21-S_ cohort, respectively, had low birth weight (<2,500g), compared to <6% in the control group (Table 1).

**Table 1:** Baseline demographics and clinical characteristics after matching.

Characteristic	cCMV_90_ cohort	Controls	cCMV_21-S_ cohort	Controls
	N (%)	N (%)	N (%)	N (%)
Observability				
1–365 days of life	54 (100.0)	3,240 (100.0)	24 (100.0)	1,440 (100.0)
366–730 days of life	34 (63.0)	2,040 (63.0)	15 (62.5)	900 (62.5)
Gender				
Male	30 (55.6)	1,800 (55.6)	15 (62.5)	900 (62.5)
Female	24 (44.4)	1,440 (44.4)	9 (37.5)	540 (37.5)
Birth quarter				
Q1	11 (20.4)	660 (20.4)	6 (25.0)	360 (25.0)
Q2	14 (25.9)	840 (25.9)	7 (29.2)	420 (29.2)
Q3	14 (25.9)	840 (25.9)	<5 (-)	180 (12.5)
Q4	15 (27.8)	900 (27.8)	8 (33.3)	480 (33.3)
Birth weight				
Extremely low (P07.0-)	<5 (-)	11 (0.34)	<5 (-)	<5 (-)
Low (P07.1-)	14 (25.9)	128 (4.0)	12 (50.0)	74 (5.1)
High (P08.1 or P08.2)	0 (0.0)	19 (0.6)	0 (0.0)	8 (0.6)
Normal (infants with none of the stated codes)	37 (68.5)	3,082 (95.1)	9 (37.5)	1,357 (94.2)
Region				
North	10 (18.5)	574 (17.7)	<5 (-)	221 (15.3)
East	9 (16.7)	343 (10.6)	7 (29.2)	149 (10.3)
West	21 (38.9)	1,171 (36.1)	8 (33.3)	534 (37.1)
South	14 (25.9)	1,145 (35.3)	7 (29.2)	533 (37.0)
Unknown	0 (0.0)	7 (0.2)	0 (0.0)	<5 (-)

Baseline demographics and clinical characteristics were assessed during the first 1–365 days of life.

cCMV, congenital cytomegalovirus; cCMV_90_, infants with cCMV diagnosis during the first 90 days of life; cCMV_21-S_, infants with inpatient cCMV diagnosis and symptoms during the first 21 days of life; Controls, infants with no cCMV or CMV diagnosis in the observation period; Q1, January 1^st^–March 31^st^; Q2, April 1^st^–June 30^th^; Q3, July 1^st^–September 30^th^; Q4, October 1^st^–December 31^st^; North, Schleswig-Holstein, Hamburg, Bremen, Lower Saxony, Mecklenburg-Western Pomerania; East, Thuringia, Brandenburg, Berlin, Saxony, Saxony-Anhalt; West, North Rhine-Westphalia, Saarland, Rhineland-Palatinate, Hesse; South, Bavaria, Baden-Wuerttemberg.

### cCMV-associated sequelae

**Fig 2:**
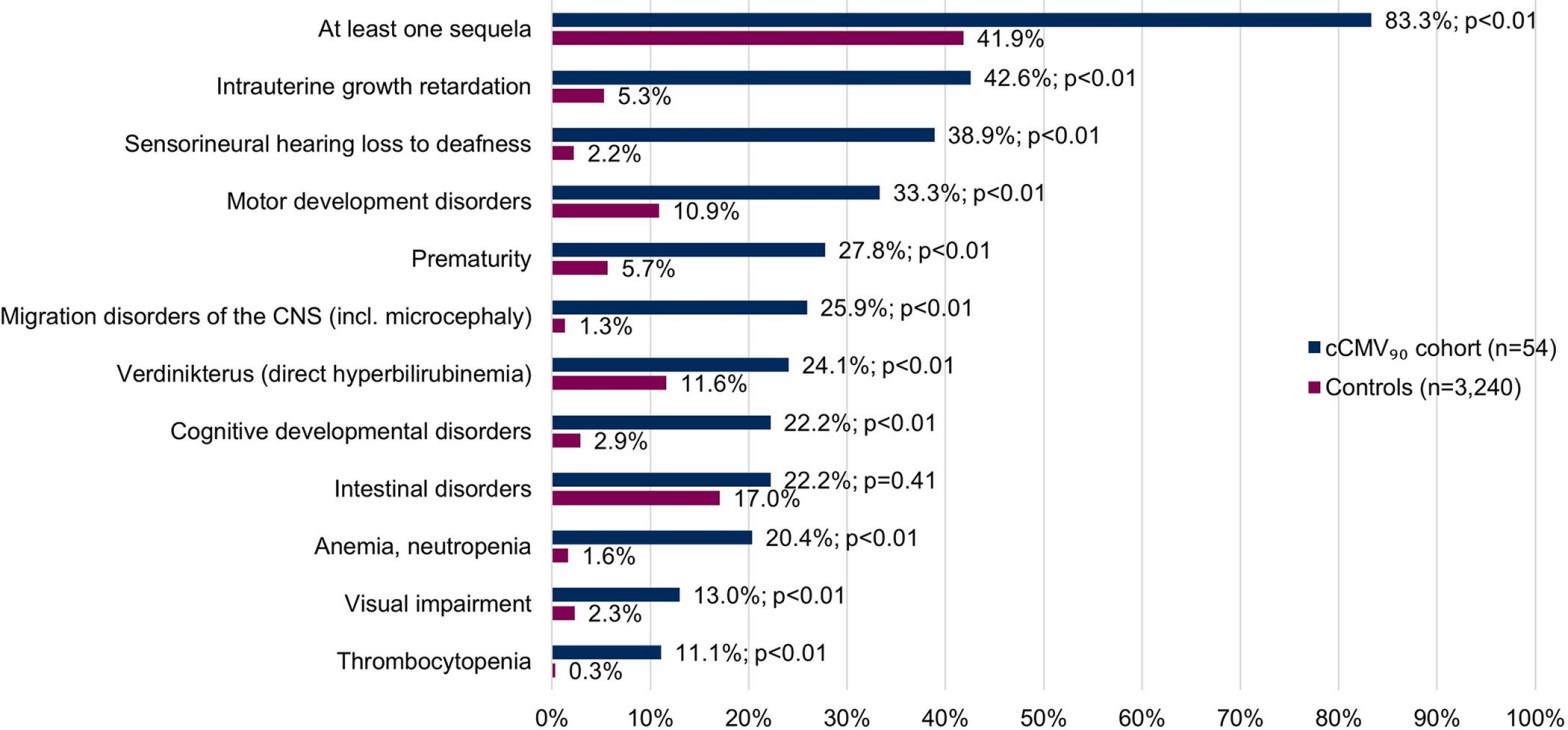
Proportions of infants with predefined sequelae present in n≥5 infants in cCMV_90_ cohort– 1–365 days. P-value <0.05 was considered as statistically significant (Mantel–Haenszel matched-pairs test). cCMV, congenital cytomegalovirus; cCMV_90_, infants with cCMV diagnosis during the first 90 days of life; Controls, infants with no cCMV or CMV diagnosis in the observation period; CNS, central nervous system; incl, including.

In the first year of life, significantly more infants in the cCMV_90_ cohort presented with at least one of the pre-defined cCMV-specific sequelae compared to the control group (83.3% vs. 41.9%, p<0.01) (Fig 2; S8 Table). The most frequent sequelae in the cCMV_90_ cohort compared to the controls were intrauterine growth retardation (42.6% vs. 5.3%, p<0.01), sensorineural hearing loss to deafness (38.9% vs. 2.2%, p<0.01), and motor development disorders (33.3% vs. 10.9%, p<0.01). Furthermore, statistically significantly more infants in the cCMV_90_ cohort were identified with visual impairment (13.0% vs. 2.3%, p<0.01).

**Fig 3:**
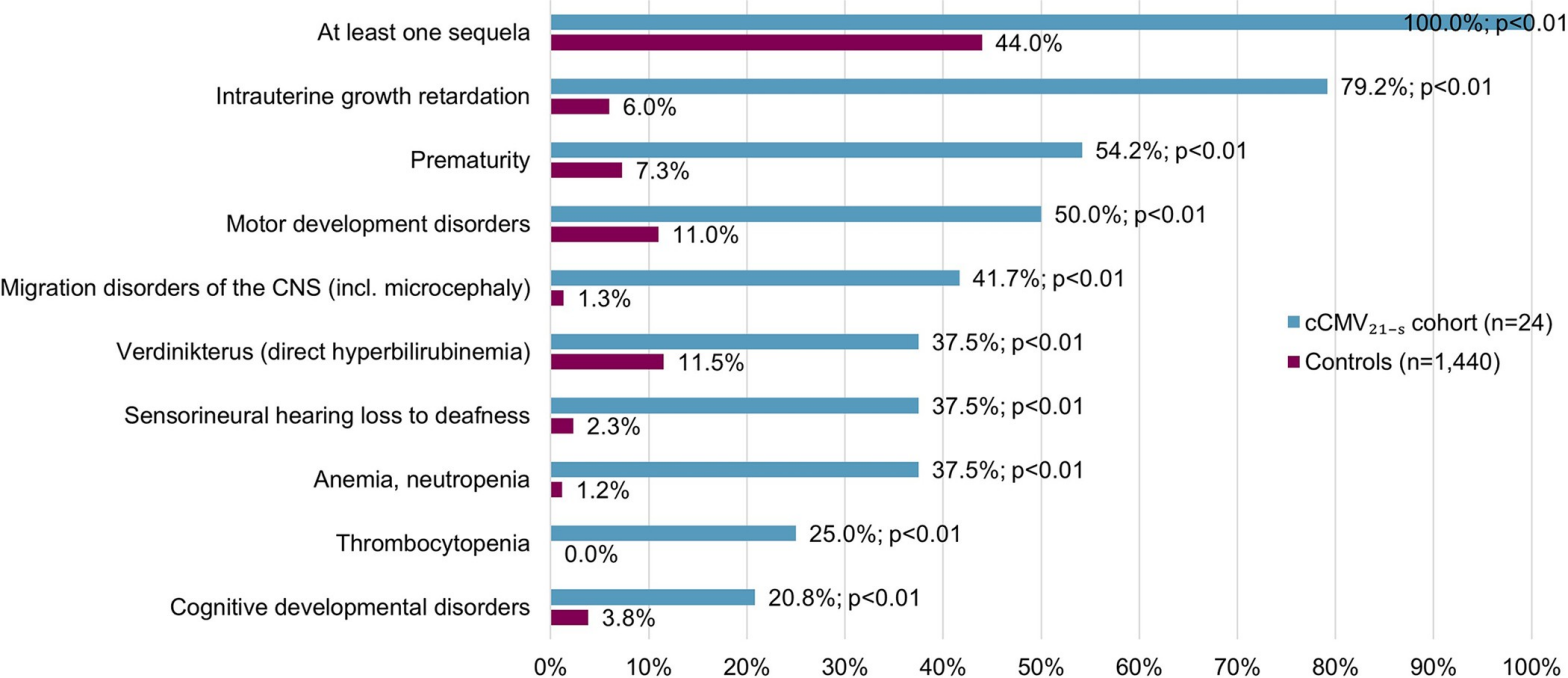
Proportions of infants with predefined sequelae present in n≥5 infants in cCMV_21-S_ cohort– 1–365 days. P-value <0.05 was considered as statistically significant (Mantel–Haenszel matched-pairs test). cCMV, congenital cytomegalovirus; cCMV_21-S_, infants with inpatient cCMV diagnosis and symptoms during the first 21 days of life; Controls, infants with no cCMV or CMV diagnosis in the observation period; CNS, central nervous system; incl, including.

Infants in the cCMV_21-S_ cohort had per definition at least one sequela during the first 21 days of life (Fig 3; S8 Table). The most frequent sequelae in the cCMV_21-S_ cohort compared to the controls during the first year of life were intrauterine growth retardation (79.2% vs. 6.0%, p<0.01), prematurity (54.2% vs. 7.3%, p<0.01), and motor development disorders (50.0% vs. 11.0%, p<0.01). Furthermore, statistically significantly more infants in the cCMV_21-S_ cohort were identified with sensorineural hearing loss to deafness (37.5% vs. 2.3%, p<0.01).

### Healthcare resource use

#### Hospitalizations

Almost all infants in the cCMV_90_ cohort (98.1%) were at least once hospitalized during their first year of life (Table 2). The two most frequent 3-digit ICD-10-GM primary diagnoses (highest percentage of infants with at least one code) in the cCMV_90_ cohort with significant differences to their controls were disorders of newborn related to short gestation and low birth weight (P07, 29.6% vs. 4.7%, p<0.01), and conductive and sensorineural hearing loss (H90, 16.7% vs. 0.7%, p<0.01). Additionally, 70.4% of infants in the cCMV_90_ cohort were hospitalized at least once for congenital viral diseases, incl. cCMV (P35) and 20.4% of infants for cytomegaloviral disease (B25) (see S9 Table).

**Table 2:** Hospitalizations and outpatient physician visits during the first 1–365 days of life.

	cCMV_90_ cohort	Controls	Mean difference	cCMV_21-S_ cohort	Controls	Mean difference
			(CI)			(CI)
No. of patients with hospitalizations						
n (%)	53 (98.1)	1,118 (34.5)		24 (100.0)	479 (33.3)	
P-value ^a^	<0.01			<0.01		
Frequency (based on total number of patients)						
Mean	4.4	0.6	3.7 (2.8–4.6)	5.7	0.6	5.1 (3.6–6.5)
SD	3.3	1.3		3.7	1.3	
Min	0.0	0.0		1.0	0.0	
Q1	2.0	0.0		3.0	0.0	
Median	3.0	0.0		4.5	0.0	
Q3	6.8	1.0		9.0	1.0	
Max	13.0	14.0		13.0	17.0	
P-value ^a^	<0.01			<0.01		
Length of stay (based on patients with hospitalizations)						
Mean	30.3	9.0	21.2 (10.4–32.0)	46.5	9.3	37.2 (18.8–55.5)
SD	40.5	17.8		45.8	15.6	
Min	1.0	1.0		6.0	1.0	
Q1	5.0	2.0		17.8	2.0	
Median	13.0	4.0		29.0	4.0	
Q3	48.0	8.0		65.5	9.5	
Max	208.0	244.0		208.0	209.0	
P-value ^a^	<0.01			<0.01		
No. of patients with outpatient visits						
n (%)	54 (100.0)	3,230 (99.7)		24 (100.0)	1,434 (99.6)	
P-value ^a^	0.68			0.75		
Frequency (based on total number of patients)						
Mean	27.7	14.1	13.6 (9.6–17.6)	30.3	14.1	16.1 (9.5–22.7)
SD	15.0	7.0		16.4	6.9	
Min	5.0	0.0		5.0	0.0	
Q1	15.5	10.0		17.5	10.0	
Median	25.5	13.0		29.5	13.0	
Q3	37.5	17.0		39.3	17.0	
Max	68.0	82.0		68.0	62.0	
P-value ^a^	<0.01			<0.01		

^a^ P-value <0.05 was considered as statistically significant (Wilcoxon rank-sum test for continuous variables and Mantel–Haenszel matched-pairs test for dichotomous variables).

cCMV, congenital cytomegalovirus; cCMV_90_, infants with cCMV diagnosis during the first 90 days of life; cCMV_21-S_, infants with inpatient cCMV diagnosis and symptoms during the first 21 days of life; Controls, infants with no cCMV or CMV diagnosis in the observation period; CI, 95% confidence interval; SD, standard deviation; Min, minimum; Q1, 25^th^ percentile; Q3, 75^th^ percentile; Max, maximum; No, number.

All infants in the cCMV_21-S_ cohort presented with a cCMV-related hospitalization (ICD-10-GM code P35.1 as primary or secondary diagnoses) during the first 21 days of life as this was part of the inclusion criteria for this cohort (Table 2). The two most common 3-digit ICD-10-GM primary diagnoses in the cCMV_21-S_ cohort with significant differences to their controls were for disorders related to prematurity and low birthweight (P07, 58.3% vs. 5.4%, p<0.01) and conductive and sensorineural hearing loss (H90, 25.0% vs. 0.6%, p<0.01). Additionally, 58.3% of all infants in the cCMV_21-S_ cohort were hospitalized at least once for congenital viral diseases, incl. cCMV (P35), and 37.5% of infants for cytomegaloviral disease (B25) (see S9 Table).

Infants in the cCMV_90_ cohort had, on average, 7.3 times more hospitalizations (4.4 vs. 0.6 hospitalizations, p<0.01) and infants in cCMV_21-S_ cohort had 9.5 times more hospitalizations (5.7 vs. 0.6 hospitalizations, p<0.01) compared to their controls. Hospitalized infants with cCMV stayed, on average, significantly longer in hospital. Mean length of stay was 30.3 days in cCMV_90_ cohort (vs. 9.0 days, p<0.01) and 46.5 days in cCMV_21-S_ cohort (vs. 9.3 days in controls, p<0.01) (Table 2).

#### Outpatient care

Infants in both cCMV cohorts had more outpatient physician visits compared to their respective controls during the first year of life. On average, infants in cCMV_90_ cohort had 2.0 times more outpatient physician visits (27.7 vs. 14.1 visits, p<0.01) and infants in the cCMV_21-S_ cohort 2.1 times more visits (30.3 vs. 14.1 visits, p<0.01) than the control group (Table 2).

The most frequently documented outpatient ICD-10-GM diagnoses in both cCMV cohorts and the control group did not differ significantly, as these were related to encounters for general examination or vaccination.

The three most common ICD-10-GM diagnoses recorded in the outpatient setting with significant differences between the cCMV_90_ cohort and the controls were for disorders of refraction and accommodation (H52, 33.3% vs. 6.8%, p<0.01), other and unspecified infectious diseases (B99, 29.6% vs. 18.1%, p<0.01), and specific developmental disorder of motor function (F82, 25.9% vs. 9.9%, p<0.01). Additionally, cytomegaloviral disease (B25) was recorded in 53.7% of infants, and congenital viral disease, incl. cCMV (P35) in 44.4% of infants.

In the cCMV_21-S_ cohort, the three most common outpatient ICD-10-GM diagnoses with significant differences compared to the controls were for disorders of newborns related to short gestation and low birth weight (P07, 45.8% vs. 5.7%, p<0.01), disorders of refraction and accommodation (H52, 37.5% vs. 6.0%, p<0.01), and specific developmental disorder of motor function (F82, 37.5% vs. 9.9%, p<0.01). Additionally, cytomegaloviral disease (B25) was recorded in 62.5% of infants, and congenital viral diseases, incl. cCMV (P35) in 50.0% of infants.

#### Outpatient pharmaceuticals

Substances (identified by ATC codes) with statistically significant differences regarding number of infants receiving respective outpatient prescriptions in the cCMV_90_ cohort compared to the control group were valganciclovir (35.2% vs. 0.0%, p<0.01), iron(II)glycinsulfat (20.4% vs. 2.3%, p<0.01), ipratropiumbromide (9.3% vs. 2.6%, p = 0.01), and nystatine (9.3% vs. 1.5%, p<0.01) (Table 3).

**Table 3:** Proportions of infants with at least one respective outpatient prescription^a^ during 1–365 days of life.

ATC code	Substance	cCMV_90_ cohort		Controls			cCMV_21-S_ cohort		Controls		
		n	%	n	%	p-value ^b^	n	%	n	%	p-value ^b^
A01 Stomatological preparations											
A01AA51	Sodium fluorides, combinations	10	18.5	488	15.1	0.53	5	20.8	229	15.9	0.64
A01AB09	Miconazole	7	13.0	269	8.3	0.33	<5	/	116	8.1	/
A03 Drugs for functional gastrointestinal disorders											
A03AX13	Silicones	16	29.6	632	19.5	0.09	8	33.3	276	19.2	0.14
A07 Antidiarrheals, intestinal anti-inflammatory/anti-infective agents											
A07AA02	Nystatin	5	9.3	49	1.5	<0.01	<5	/	20	1.4	/
A11 Vitamins											
A11CC	Vitamin D and analogues	12	22.2	535	16.5	0.29	5	20.8	217	15.1	0.57
A11CC05	Colecalciferol	31	57.4	1,909	58.9	0.93	13	54.2	830	57.6	0.89
A11CC55	Colecalciferol, combinations	9	16.7	547	16.9	0.96	<5	/	246	17.1	/
B03 Antianemic preparations											
B03AA01	Iron(II) glycine sulfate	11	20.4	74	2.3	<0.01	7	29.2	35	2.4	<0.01
D01 Antifungals for dermatological use											
D01AA20	Antibiotic combinations	9	16.7	310	9.6	0.13	6	25.0	123	8.5	0.01
D02 Emollients and protectives											
D02AB	Zinc-containing medicines	5	9.3	231	7.1	0.74	<5	/	111	7.7	/
J05 Antivirals for systemic use											
J05AB14	Valganciclovir	19	35.2	0	0.0	<0.01	12	50.0	0	0.0	<0.01
M01 Antiinflammatory and antirheumatic products											
M01AE01	Ibuprofen	12	22.2	988	30.5	0.24	<5	/	431	29.9	/
N02 Analgesics											
N02BE01	Paracetamol	44	81.5	2,636	81.4	0.98	19	79.2	1,174	81.5	0.98
R01 Nasal preparations											
R01AA07	Xylometazoline	29	53.7	1,797	55.5	0.90	13	54.2	799	55.5	0.90
R03 Drugs for obstructive airway diseases											
R03AC02	Salbutamol	13	24.1	459	14.2	0.06	5	20.8	187	13.0	0.41
R03BB01	Ipratropium bromide	5	9.3	85	2.6	0.01	<5	/	28	1.9	/
R05 Cough and cold preparations											
R05CA12	Casein hydrolysate	6	11.1	539	16.6	0.37	<5	/	230	16.0	/
R05CB06	Ambroxol	8	14.8	435	13.4	0.92	5	20.8	203	14.1	0.52
S01 Ophthalmologicals											
S01AA11	Gentamicin	7	13.0	237	7.3	0.19	<5	/	106	7.4	/
S01AE01	Ofloxacin	9	16.7	321	9.9	0.16	<5	/	139	9.7	/
V07 All other non-therapeutic products											
V07AB	Solvents and thinners, including rinsing solutions	9	16.7	346	10.7	0.24	<5	/	158	11.0	/

^a^ Only substances which were prescribed for at least 5 infants in cCMV_90_ are displayed.

^b^ P-value <0.05 was considered as statistically significant (Mantel–Haenszel matched-pairs test).

cCMV, congenital cytomegalovirus; cCMV_90_, infants with cCMV diagnosis during the first 90 days of life; cCMV_21-S_, infants with inpatient cCMV diagnosis and symptoms during the first 21 days of life; Controls, infants with no cCMV or CMV diagnosis in the observation period; ATC, Anatomical Therapeutic Chemical.

In the cCMV_21-S_ cohort, these were valganciclovir (50.0% vs. 0.0%, p<0.01), iron(II)glycinsulfat (29.2% vs. 2.4%, p<0.01), and antibiotic combinations (25.0% vs. 8.5%, p<0.01) (Table 3).

#### Physical and occupational therapy prescriptions

A share of 31.8% of infants in the cCMV_90_ cohort (vs.12.8%, p<0.01) and 42.9% in the cCMV_21-S_ cohort (vs. 13.2%, p<0.01) received at least one prescription for physical and occupational therapy. Infants in the cCMV_90_ cohort received, on average, four times more prescriptions (1.2 vs. 0.3 prescriptions, p<0.01) and infants in cCMV_21-S_ cohort 6.3 times more prescriptions (1.9 vs. 0.3 prescriptions, p<0.01) than their controls (Table 4).

**Table 4:** Physical and occupational therapy prescriptions during the first 1–365 days of life.

	cCMV_90_ cohort	Controls	Mean difference	cCMV_21-S_ cohort	Controls		Mean difference
			(CI)				(CI)
No. of patients with physical and occupational therapies				
n (%)	14 (31.8)	283 (12.8)		9 (42.9)	141 (13.2)		
P-value ^a^	<0.01			<0.01			
Frequency (based on total number of patients)							
Mean	1.2	0.3	0.9 (0.2–1.5)	1.9	0.3		1.6 (0.4–2.8)
SD	2.2	1.2		2.8	1.1		
Min	0.0	0.0		0.0	0.0		
Q1	0.0	0.0		0.0	0.0		
Median	0.0	0.0		0.0	0.0		
Q3	1.3	0.0		4.0	0.0		
Max	8.0	15.0		8.0	11.0		
P-value ^a^	<0.01			<0.01			

^a^ P-value <0.05 was considered as statistically significant (Wilcoxon rank-sum test for continuous variables and Mantel–Haenszel matched-pairs test for dichotomous variables).

Due to missing data, results for physical and occupational therapy prescriptions are based on n = 44 infants in cCMV_90_ cohort and n = 21 infants in cCMV_21-S_ cohort with valid data for physical and/or occupational therapies.

cCMV, congenital cytomegalovirus; cCMV_90_, infants with cCMV diagnosis during the first 90 days of life; cCMV_21-S_, infants with inpatient cCMV diagnosis and symptoms during the first 21 days of life; Controls, infants with no cCMV or CMV diagnosis in the observation period; CI, 95% confidence interval; SD, standard deviation; Min, minimum; Q1, 25^th^ percentile; Q3, 75^th^ percentile; Max, maximum; No = number.

## Discussion

During the first year of life, more than 80% of infants with a cCMV diagnosis in our study presented with at least one cCMV-associated sequela, with intrauterine growth retardation, sensorineural hearing loss to deafness, and motor development disorders being the most frequently observed diagnoses. Further, infants with cCMV were statistically more frequently hospitalized and had more outpatient physician visits compared to infants without cCMV.

Nevertheless, there is limited awareness and knowledge of cCMV infection in everyday clinical practice [24–26], which may lead to an underestimation of cCMV burden in Germany. Even when typical cCMV symptoms are present, cCMV infection may not always be considered by physicians as a potential cause and therefore no further diagnostics for cCMV may be performed [27]. Based on the results of our study, intrauterine growth retardation was the most frequent sequela in both the cCMV_90_ and cCMV_21-S_ cohort (42.6% and 79.2%, respectively), which may provide an important clue to cCMV infection. It may be reasonable to suggest testing for cCMV infection in infants with unexplained intrauterine growth retardation at birth or prematurity [28]. Targeted CMV testing among newborns who failed newborn hearing screening, has been explored by different study groups [29, 30]. The study by Fowler et al. [29] evaluated a group of newborns specifically screened for CMV, who underwent newborn hearing screening in the follow-up. They found that among 443 newborns with positive CMV test result, 20 (4.5%) newborns with sensorineural hearing loss could be identified via newborn hearing screening. Considering only the 35 CMV-positive infants with sensorineural hearing loss, 20 (57%) of newborns with CMV-related sensorineural hearing loss were identified via newborn hearing screening. Although newborn hearing screening may not be able to identify all newborns with CMV-related sensorineural hearing loss and by the time hearing loss is confirmed by diagnostic audiologic evaluation it may be too late to confirm cCMV, targeted CMV testing in those infants with unexplained hearing loss might be a supportive measure to identify CMV-related impairments in infants. Also, less common diagnoses such as visual impairment may be an important indication for cCMV infection. For instance, visual impairment was diagnosed in about 13% of the identified cCMV infants. Even though this was not amongst the most frequently identified sequelae, it may comprise a substantial clinical burden to the infants in the long-term as many of the affected infants require ongoing care and special therapeutic and educational services, which lead to considerable healthcare burden [31]. In general, there is a necessity for more awareness of cCMV infection in newborns and associated complications.

Generally, our results are in line with the sparse information currently available in the literature. In our study, sensorineural hearing loss was diagnosed in around 39% of infants with cCMV during the first year of life, which is within the range of findings from other studies (32%-41% in symptomatic cCMV infants) [32]. Our results suggest that infants diagnosed with cCMV are likely to suffer from more than one sequela during the first year of life, which has also been reported by Modrow and colleagues [23].

However, not all infants with a cCMV diagnosis showed a record for any of the pre-defined cCMV-specific sequelae and not all pre-defined sequelae reported in the literature like optic atrophy, chorioretinitis, pneumonia, and abnormal findings in cerebrospinal fluid, were identified in our cCMV cohorts [23]. Nevertheless, it may be reasonable to assume that all infants with a confirmed cCMV diagnosis in our dataset might have been symptomatic in one way or another. We hypothesize that all infants must have been detected and diagnosed after targeted investigation, possibly triggered by signs or symptoms in the infant’s development suspicious for cCMV infection, as to date no national screening program for cCMV exists in Germany. There are two possible explanations for the lack of recorded symptoms in these infants: 1) Frequently, only ICD-10-GM codes that are relevant for reimbursement purposes are coded by the physician and it might be possible that physicians did not record all symptoms (ICD-10-GM codes), as they might have not been relevant for reimbursement; 2) we assessed the occurrence of pre-defined ICD-10-GM codes based on clinical expertise and published literature [23], the list of relevant diagnoses, however, could be incomplete. In both cases the burden of cCMV sequelae reported in this study might underestimate the true clinical burden.

Depending on the definition of infants with cCMV used in this study, the reported frequency of sequelae and differences to the control group vary by comparison. The decision to use two alternative definitions for cCMV was based on the uncertainty associated with the attempt to retrospectively distinguish infants with cCMV infection from infants with postnatal CMV infection solely based on claims data information [6].

Moreover, this study focused on the first year of life, even though some sequelae will affect the infants for the rest of their life and some sequelae of previously asymptomatic infants might be detected later in life, such as sensorineural hearing loss and cognitive impairment [32]. Delayed onset hearing loss, for instance, has been reported to occur in about 7–10% of initially asymptomatic infants at a median age of 44 months [33]. Due to potential late-onset of hearing impairments, it is possible that these may be missed by universal hearing screening [28, 34] and even if detected, the association between hearing loss and cCMV infection may no longer be identifiable at this point. Even though a timely diagnosis of cCMV infection in symptomatic as well as asymptomatic newborns is crucial for early interventions with improved chances of reducing the risk of severe and permanent disabilities [23, 25], to date, national newborn screening for cCMV is not established in Germany.

The resource use associated with management and treatment of cCMV infection and associated sequelae, especially in the first year of life, was barely assessed so far. In a retrospective cohort study from Israel, infants with cCMV were nearly twice as likely to have a hospital admission compared to their controls (8.5% cases vs 4.5% control; p<0.001) [35]. A retrospective administrative health insurance claims database analysis from the US compared HCRU and costs between infants with cCMV diagnosis at birth and during the first year of life versus matched infants without diagnosed cCMV [36]. The study found that around 32% of the cCMV infants had at least one all-cause hospitalization during the 12-month follow-up period. Almost all infants with cCMV in our study had been hospitalized at least once during the first year of life. This remarkably higher proportion can be explained by the fact that birth itself constitutes one important reason for hospitalization in the first year of life. Even though both studies included the day of birth in the follow-up period, in the US, birth-related claims may have been included on a mother’s insurance policy [36], whereas in Germany each newborn, theoretically, has its own insurance coverage beginning with the birthday.

Additionally, in our study we observed twice as many outpatient physician visits in the cCMV cohorts compared to their controls. One factor contributing to these excess outpatients visits might be follow-up visitis for check-up and associated pharmaceutical treatments such as valganciclovir or ganciclovir. Currently, there are still no approved drugs for cCMV treatment in infants [31]. Newborns with symptomatic cCMV infection may receive (currently off-label use) antiviral therapy with valganciclovir or ganciclovir depending on disease severity and presence of symptoms [9, 23, 33, 37]. The observed outpatient prescriptions of valganciclovir in the cCMV_90_ and the cCMV_21-S_ cohorts (35.2% and 50.0%, respectively) indicate that there is need for the treatment of cCMV to reduce viral burden and the risk of severe disabilities in these infants. It should be considered that the actual number of infants receiving antiviral therapy might be underestimated in our study as due to the nature of this database, most pharmaceuticals dispensed in the inpatient setting are covered as lump sums by the German Diagnosis Related Group (G-DRG) reimbursement system and are therefore not separately identifiable in German claims data records.

Additional limitations of our study are associated with difficulties in the retrospective identification of infants with cCMV (e.g., dried blood spots are destroyed in Germany 90 days after birth) [6, 38], as well as special challenges concerning the identification of cCMV in electronic health records [39, 40]. Claims data are primarily collected for reimbursement purposes, i.e., the database includes only information on infants who have seen a physician and caused reimbursement for the health insurance. Moreover, clinical or laboratory results are not available in the database. Therefore, differentiation between virologically confirmed diagnoses and other (clinical) reasons for entry of cCMV records is not available. Consequently, the classification of infants in the cCMV cohorts depends to some extent on the ICD coding behavior of physicians, which, as common for secondary data analyses, was beyond our control. Data are anonymized for data protection reasons, so validation of diagnoses against medical charts is not possible. At present, there are also no additional validated tools available to specifically identify infants with cCMV in German claims databases. Therefore, we established a detailed identification process for infants with cCMV. In the case definition, no specific symptoms for the cCMV_90_ cohort were required. However, as there is no universal newborn screening for cCMV in Germany, we assume that infants in the cCMV_90_ cohort must have been detected and diagnosed after targeted investigation, possibly triggered by signs or symptoms in their development, so that they were symptomatic to some extent, i.e., asymptomatic cCMV or misdiagnosed symptomatic infants are likely not covered by our study population. In this context, the actual burden of cCMV in Germany may be underestimated, since undiagnosed patients as well as services that are not reimbursed by the SHI (e.g. certain individual health services) have not been captured in the database. Also infants in the control group may not have been strictly investigated for cCMV typical sequelae resulting in an underestimation of those diagnoses in the control group. This bias might have induced an increased difference between cases and controls.

Considering the presumably underestimated frequencies of sequelae from this study, it is essential to understand the actual burden of sequelae and HCRU in newborns with cCMV infection. A systematic collection of information (by physicians and laboratories) would offer the opportunity to produce reliable numbers on the actual burden of cCMV and associated sequelae in newborns in Germany and finally, to diagnose and manage potential cCMV infections as early as possible.

## Conclusion

In Germany, the burden of sequelae in the first year of life in children diagnosed with cCMV is high, with significant differences compared to children without cCMV diagnosis. More than 80% of all identified infants with cCMV diagnosed during the first 90 days of life suffer from at least one sequela during the first year of life, which include also long-term disabilities such as sensorineural hearing loss to deafness and visual impairment. The potential disease and healthcare burden in infants with cCMV diagnoses calls for preventative steps against cCMV infection and a comprehensive monitoring of cCMV and its sequelae in Germany.

## Supporting information

S1 File Case definition. (DOCX)

S1 Table Proportions of infants with predefined sequelae during the first 366–730 days of life. (DOCX)

S2 Table Hospitalizations and outpatient physician visits during the first 366–730 days of life. (DOCX)

S3 Table Proportions of infants with reasons for hospitalizations during the first 366–730 days of life. (DOCX)

S4 Table Proportions of infants with at least one respective outpatient prescription during the first 366–730 days of life. (DOCX)

S5 Table Physical and occupational therapy prescriptions during the first 366–730 days of life. (DOCX)

S6 Table Exclusion criteria for overall study population. (DOCX)

S7 Table cCMV-specific symptoms and sequelae. (DOCX)

S8 Table Proportions of infants with predefined sequelae during the first 1–365 days of life. (DOCX)

S9 Table Proportions of infants with reasons for hospitalizations during the first 1–365 days of life. (DOCX)
