# Supplementary material for: Burden of sequelae and healthcare resource utilization in the first year of life in infants born with congenital cytomegalovirus (cCMV) infection in Germany: A retrospective statutory health insurance claims database analysis
Source: PLoS One. 2023 Nov 16;18(11):e0293869. doi: 10.1371/journal.pone.0293869 (PMC10653416; doi:10.1371/journal.pone.0293869)
Supplement: S1 File — (DOCX) [file pone.0293869.s001.docx]

# S1 File. Case definition

To establish criteria which appropriately define the target study population, i.e., infants with congenital cytomegalovirus (cCMV), using German claims data, a case definition was developed based on clinical expertise in combination with published literature.

All newborns in the Institute for Applied Health Research Berlin (InGef) Database from 2014-2018 were considered for this analysis. For the assessment of the first year of life (*second year of life*) infants born in 2014-2018 (*born in 2014-2017*), needed to be continuously observable for at least 365 days of life (*730 days of life*) including birth date, except for infants who deceased in the follow-up period. For the outcome analyses of the identified infants, claims data from 2014-2019 was used.

From all identified newborns, infants with International Statistical Classification of Diseases and Related Health Problems, 10^th^ Revision, German Modification (ICD-10-GM) records of defined, immunocompromised diseases, which may increase the risk of postnatal CMV infection (see S1 Table) were excluded.

All remaining infants served as the overall study population from which the congenital cytomegalovirus (cCMV) cohorts as well as the two control groups were drawn.

The patient selection process is described in detail in the following.

## cCMV cohorts

From the identified newborns, two cohorts of infants with cCMV were identified. The following steps were applied to identify infants in cCMV-cohort 1 (cCMV_90_):

**Step 1**: all infants with an ICD-10-GM diagnosis record for cCMV (P35.1) in the inpatient sector (primary or secondary diagnosis) or outpatient sector (verified or suspected diagnosis) in the first 365 days were identified.

**Step 2**: of these, all infants were identified if they had

1. an inpatient diagnosis (primary or secondary diagnosis) for cCMV (P35.1) during any hospital stay that began in the first 90 days of life (including birth date).

***or***

1. an outpatient diagnosis (verified or suspected diagnosis) for cCMV (P35.1) within the first 90 days of life. As outpatient diagnoses in Germany are only available on a quarterly basis, the day-specific records of Official German Remuneration Scheme for Outpatient Care (Einheitlicher Bewertungsmaßstab, EBM) codes were used as a proxy to estimate the date of the outpatient diagnosis. If an EBM code was recorded within the 90-days window by the same physician who had recorded the verified or suspected cCMV diagnosis, the cCMV diagnosis was assumed to be recorded within the 90 days. The EBM code und the ICD-10-GM code were linked by the physician’s lifelong identification number (Lebenslange Arztnummer, LNAR).

To increase validity of outpatient diagnoses recorded within the 90 days window, the included outpatient (verified and suspected) diagnoses were only considered if they could be validated with a second cCMV diagnosis in the follow-up period. To this end, the infants were only further included if they had at least another inpatient (primary or secondary diagnosis) or at least one more outpatient (verified diagnosis only) cCMV diagnosis in another quarter or by another physician after the first (initial) outpatient diagnosis.

- The identified infants with an ICD-10-GM record for cCMV (P35.1) during an inpatient stay that began during the first 90 days of life (including birth) or with an outpatient diagnosis that could be linked to the first 90 days of life and validated with a second diagnosis were defined as cCMV cohort 1 (cCMV_90_)

Further steps were applied to identify infants in cCMV-cohort 2 (cCMV_21-S_):

**Step 3**: all infants with a hospital admission during the first 21 days of life (including birth) were identified.

**Step 4**: of these, all infants with an inpatient diagnosis (primary or secondary diagnosis) for cCMV (P35.1) during any hospital admission in this 21-day window were identified.

**Step 5**: of these, all infants with an inpatient diagnosis (primary or secondary diagnosis) of at least one pre-defined cCMV-specific symptom (based on clinical expertise and published literature^[[1]](#footnote-2)^) (S 2 Table) during any hospital admission in this 21-day window were identified.

- The identified infants with cCMV diagnosis and specific symptoms during a hospital admission in their first 21 days of life (including birth) were defined as cCMV cohort 2 (cCMV_21-S_).

For the identification of cCMV we used ICD-10-GM codes, which is the official classification for the encoding of diagnoses in inpatient and outpatient medical care in Germany since 2000. Clinicians in the outpatient setting are required to add one of the following specifications to the ICD-10-GM codes: “suspected diagnosis”, “diagnosis ruled out”, “condition post diagnosis”, or “verified diagnosis”. For instance, “suspected” may be coded if the physician is not certain about the presence of the coded disease and a confirming laboratory analysis is still pending. To ensure the accuracy of cCMV diagnoses, only verified diagnoses and suspected diagnoses in the respective time frames were included. Outpatient verified and suspected diagnoses were only considered if they were verified with an additional inpatient (primary or secondary) cCMV diagnosis or at least one more outpatient (verified diagnosis only) cCMV diagnosis in another quarter or by another physician after the first (initial) outpatient diagnosis.

## Control group

From the overall study population, all infants with an outpatient diagnosis (verified diagnosis) or an inpatient diagnosis (primary or secondary diagnosis) for cCMV (P35.1) or CMV (B25) at any time in their observation period were excluded. Infants with B25 diagnosis were excluded to decrease the probability of including infants with cCMV in the control group that might have been misdiagnosed with (postnatal) CMV. Out of the remaining infants from the overall study population, infants without cCMV (P35.1) or CMV (B25) ICD-10-GM codes were defined as the control group. As infants in the control group needed to be continuously observable for at least as long as their matched cCMV cases, controls who deceased before the cases were excluded.

1. Modrow S, et al., *Management der kongenitalen Zytomegalievirus-Infektion bei Neugeborenen.* Kinder- und Jugendarzt, 2018(49): p. 107-117. [↑](#footnote-ref-2)
