# Supplementary material for: Burden of sequelae and healthcare resource utilization in the first year of life in infants born with congenital cytomegalovirus (cCMV) infection in Germany: A retrospective statutory health insurance claims database analysis
Source: PLoS One. 2023 Nov 16;18(11):e0293869. doi: 10.1371/journal.pone.0293869 (PMC10653416; doi:10.1371/journal.pone.0293869)
Supplement: S1 Table — (DOCX) [file pone.0293869.s002.docx]

S1 Table. Proportions of infants with predefined sequelae during the first 366-730 days of life.

| Sequelae | cCMV_90_ cohort | | Controls | |  | cCMV_21-S_ cohort | | Controls | |  |
| --- | --- | --- | --- | --- | --- | --- | --- | --- | --- | --- |
|  | n | % | n | % | p-value ^a^ | n | % | n | % | p-value ^a^ |
| At least one sequela | 27 | 79.4 | 880 | 43.1 | <0.01 | 14 | 93.3 | 389 | 43.2 | <0.01 |
| Abnormal findings in cerebrospinal fluid | 0 | 0.0 | 0 | 0.0 | / | 0 | 0.0 | 0 | 0.0 |  |
| Anemia, neutropenia | <5 | / | <5 | / | / | <5 | / | <5 | / | / |
| Cerebral seizures | <5 | / | 56 | 2.7 | / | 0 | 0.0 | 21 | 2.3 | 0.55 |
| Chorioretinitis including retinal scar | 0 | 0.0 | <5 | / | / | 0 | 0.0 | 0 | 0.0 |  |
| Cognitive developmental disorders | 14 | 41.2 | 254 | 12.5 | <0.01 | 7 | 46.7 | 116 | 12.9 | <0.01 |
| Disseminated petechiae | 0 | 0.0 | 7 | 0.3 | 0.73 | 0 | 0.0 | <5 | / | / |
| Intestinal disorders | 11 | 32.4 | 513 | 25.1 | 0.45 | 5 | 33.3 | 217 | 24.1 | 0.60 |
| Hepatosplenomegaly | 0 | 0.0 | <5 | / | / | 0 | 0.0 | <5 | / | / |
| Intrauterine growth retardation (decreased birth weight according to gestational age <3rd percentile) | 6 | 17.6 | 29 | 1.4 | <0.01 | 6 | 40.0 | 13 | 1.4 | <0.01 |
| Visual impairment | <5 | / | 73 | 3.6 | / | 0 | 0.0 | 31 | 3.4 | 0.99 |
| Migration disorders of the central nervous system | <5 | / | 16 | 0.8 | / | <5 | / | 7 | 0.8 | / |
| Motor development disorders | 14 | 41.2 | 127 | 6.2 | <0.01 | 9 | 60.0 | 67 | 7.4 | <0.01 |
| Optic atrophy | 0 | 0.0 | <5 | / | / | 0 | 0.0 | 0 | 0.0 |  |
| Paralysis | <5 | / | 7 | 0.3 | / | <5 | / | 7 | 0.8 | / |
| Pneumonia | 0 | 0.0 | 0 | 0.0 | / | 0 | 0.0 | 0 | 0.0 |  |
| Prematurity | 7 | 20.6 | 41 | 2.0 | <0.01 | 6 | 40.0 | 28 | 3.1 | <0.01 |
| Purpura | <5 | / | 9 | 0.4 | / | <5 | / | <5 | / | / |
| Sensorineural hearing loss to deafness (newborn hearing screening) | 8 | 23.5 | 21 | 1.0 | <0.01 | <5 | / | 18 | 2.0 | / |
| Thrombocytopenia | 0 | 0.0 | 0 | 0.0 | / | 0 | 0.0 | 0 | 0.0 |  |
| Verdinikterus (direct hyperbilirubinemia) | <5 | / | <5 | / | / | <5 | / | 0 | 0.0 | / |

^a^ P-value <0.05 was considered as statistically significant (Mantel–Haenszel matched-pairs test).

Infants could be diagnosed with more than one sequela.

cCMV, congenital cytomegalovirus; cCMV_90_, infants with cCMV diagnosis during the first 90 days of life; cCMV_21-S_, infants with inpatient cCMV diagnosis and symptoms during the first 21 days of life; Controls, infants with no cCMV or CMV diagnosis in the observation period.
