# Supplementary material for: Burden of sequelae and healthcare resource utilization in the first year of life in infants born with congenital cytomegalovirus (cCMV) infection in Germany: A retrospective statutory health insurance claims database analysis
Source: PLoS One. 2023 Nov 16;18(11):e0293869. doi: 10.1371/journal.pone.0293869 (PMC10653416; doi:10.1371/journal.pone.0293869)
Supplement: S2 Table — (DOCX) [file pone.0293869.s003.docx]

S2 Table. Hospitalizations and outpatient physician visits during the first 366-730 days of life.

|  | cCMV_90_ cohort | Controls | Mean  difference  (CI) | cCMV_21-S_ cohort | Controls | Mean  difference  (CI) |
| --- | --- | --- | --- | --- | --- | --- |
| No. of patients with hospitalizations | | | |  |  |  |
| n (%) | 19 (55.9) | 379 (18.6) |  | 10 (66.7) | 182 (20.2) |  |
| P-value ^a^ | <0.01 | |  | <0.01 | |  |
| Frequency (based on total number of patients) | | | |  |  |  |
| Mean | 1.9 | 0.3 | 1.6 (0.9-2.3) | 2.1 | 0.3 | 1.8 (0.8-2.8) |
| SD | 2.1 | 1.4 |  | 2.0 | 0.8 |  |
| Min | 0.0 | 0.0 |  | 0.0 | 0.0 |  |
| Q1 | 0.0 | 0.0 |  | 0.0 | 0.0 |  |
| Median | 1.0 | 0.0 |  | 2.0 | 0.0 |  |
| Q3 | 4.0 | 0.0 |  | 4.0 | 0.0 |  |
| Max | 6.0 | 51.0 |  | 6.0 | 7.0 |  |
| P-value ^a^ | <0.01 | |  | <0.01 | |  |
| Length of stay (based on patients with hospitalizations) | | | |  |  |  |
| Mean | 14.7 | 4.9 | 9.8 (-1.7-21.4) | 3.2 | 3.2 | 0.0 (-1.0-1.0) |
| SD | 34.2 | 17.7 |  | 1.8 | 7.0 |  |
| Min | 1.0 | 1.0 |  | 1.0 | 1.0 |  |
| Q1 | 2.5 | 1.0 |  | 2.0 | 1.0 |  |
| Median | 4.0 | 2.0 |  | 3.5 | 2.0 |  |
| Q3 | 7.0 | 4.0 |  | 4.0 | 3.0 |  |
| Max | 141.0 | 241.0 |  | 7.0 | 77.0 |  |
| P-value ^a^ | <0.01 | |  | 0.07 | |  |
| No. of patients with outpatient visits | | | |  |  |  |
| n (%) | 34 (100.0) | 2,032 (99.6) |  | 15 (100.0) | 893 (99.2) |  |
| P-value ^a^ | 0.72 | |  | 0.73 | |  |
| Frequency (based on total number of patients) | | | |  |  |  |
| Mean | 16.4 | 11.8 | 4.6 (1.2-8.0) | 15.1 | 11.6 | 3.5 (0.1-7.0) |
| SD | 10.1 | 7.0 |  | 6.8 | 6.3 |  |
| Min | 7.0 | 0.0 |  | 7.0 | 0.0 |  |
| Q1 | 9.3 | 7.0 |  | 10.0 | 7.0 |  |
| Median | 13.5 | 10.0 |  | 15.0 | 10.0 |  |
| Q3 | 21.0 | 15.0 |  | 19.0 | 15.0 |  |
| Max | 47.0 | 60.0 |  | 29.0 | 41.0 |  |
| P-value ^a^ | <0.01 | |  | 0.04 | |  |

^a^ P-value <0.05 was considered as statistically significant (Wilcoxon rank-sum test for continuous variables and Mantel–Haenszel matched-pairs test for dichotomous variables).

cCMV, congenital cytomegalovirus; cCMV_90_, infants with cCMV diagnosis during the first 90 days of life; cCMV_21-S_, infants with inpatient cCMV diagnosis and symptoms during the first 21 days of life; Controls, infants with no cCMV or CMV diagnosis in the observation period; CI, 95% confidence interval; SD, standard deviation; Min, minimum; Q1, 25^th^ percentile; Q3, 75^th^ percentile; Max, maximum; No, number.
