# Supplementary material for: Burden of sequelae and healthcare resource utilization in the first year of life in infants born with congenital cytomegalovirus (cCMV) infection in Germany: A retrospective statutory health insurance claims database analysis
Source: PLoS One. 2023 Nov 16;18(11):e0293869. doi: 10.1371/journal.pone.0293869 (PMC10653416; doi:10.1371/journal.pone.0293869)
Supplement: S3 Table — (DOCX) [file pone.0293869.s004.docx]

S3 Table. Proportions of infants with reasons^a^ for hospitalizations during the first 366-730 days of life.

| ICD-10-GM code | Description | cCMV_90_ cohort | | Controls | |  | cCMV_21-S_ cohort | | Controls | |  |
| --- | --- | --- | --- | --- | --- | --- | --- | --- | --- | --- | --- |
|  |  | n | % | n | % | p-value ^b^ | n | % | n | % | p-value ^b^ |
| P35 | Congenital viral diseases | 8 | 23.5 | 0 | 0.0 | N/A^c^ | <5 | / | 0 | 0.0 | N/A^c^ |
| H90 | Conductive and sensorineural hearing loss | 5 | 14.7 | <5 | / | / | <5 | / | <5 | / | / |
| F82 | Specific developmental disorder of motor function | 5 | 14.7 | 12 | 0.6 | <0.01 | <5 | / | <5 | / | / |
| P07 | Disorders of newborn related to short gestation and low birth weight, NEC | 5 | 14.7 | 12 | 0.6 | <0.01 | 5 | 33.3 | <5 | / | / |

^a^ Only inpatient primary ICD-10-GM diagnoses (3-digit), which were recorded for at least 5 infants in cCMV_90_ cohort during a hospitalization are displayed.

^b^ P-value <0.05 was considered as statistically significant (Mantel–Haenszel matched-pairs test).

^c^Comparison not applicable due to eligibility criteria.

cCMV, congenital cytomegalovirus; cCMV_90_, infants with cCMV diagnosis during the first 90 days of life; cCMV_21-S_, infants with inpatient cCMV diagnosis and symptoms during the first 21 days of life; Controls, infants with no cCMV or CMV diagnosis in the observation period; ICD-10-GM, International Classification of Diseases, 10^th^ Revision, German Modification; n, number; N/A, not applicable; NB, newborn; NEC, not elsewhere classified; w/o, without.
