# Supplementary material for: Burden of sequelae and healthcare resource utilization in the first year of life in infants born with congenital cytomegalovirus (cCMV) infection in Germany: A retrospective statutory health insurance claims database analysis
Source: PLoS One. 2023 Nov 16;18(11):e0293869. doi: 10.1371/journal.pone.0293869 (PMC10653416; doi:10.1371/journal.pone.0293869)
Supplement: S4 Table — (DOCX) [file pone.0293869.s005.docx]

S4 Table. Proportions of infants with at least one respective outpatient prescription^a^ during the first 366-730 days of life.

| ATC code | Substance | cCMV_90_ cohort | | Controls | |  | cCMV_21-S_ cohort | | Controls | |  |
| --- | --- | --- | --- | --- | --- | --- | --- | --- | --- | --- | --- |
|  |  | n | % | n | % | p-value ^b^ | n | % | n | % | p-value ^b^ |
| A11 Vitamins | |  |  |  |  |  |  |  |  |  |  |
| A11CC05 | Colecalciferol | 9 | 26.5 | 472 | 23.1 | 0.80 | 5 | 33.3 | 192 | 21.3 | 0.42 |
| M01 Antiinflammatory and antirheumatic products | | | | | | | | | | | |
| M01AE01 | Ibuprofen | 19 | 55.9 | 1,182 | 57.9 | 0.95 | 6 | 40.0 | 486 | 54.0 | 0.41 |
| N02 Analgesics | | | | | | | | | | | |
| N02BE01 | Paracetamol | 18 | 52.9 | 1,061 | 52.0 | 0.91 | 7 | 46.7 | 444 | 49.3 | 0.84 |
| R01 Nasal preparations | |  |  |  |  |  |  |  |  |  |  |
| R01AA07 | Xylometazoline | 23 | 67.6 | 1,210 | 59.3 | 0.42 | 10 | 66.7 | 516 | 57.3 | 0.64 |
| R03 Drugs for obstructive airway diseases | | | | | | | | | | | |
| R03AC02 | Salbutamol | 6 | 17.6 | 329 | 16.1 | 1.00 | <5 | / | 134 | 14.9 | / |
| R06 Antihistamines for systemic use | |  |  |  |  |  |  |  |  |  |  |
| R06AA02 | Diphenhydramine | 5 | 14.7 | 252 | 12.4 | 0.88 | <5 | / | 99 | 11.0 | / |
| S01 Ophthalmologicals | |  |  |  |  |  |  |  |  |  |  |
| S01AE01 | Ofloxacin | 7 | 20.6 | 271 | 13.3 | 0.32 | <5 | / | 120 | 13.3 | / |
| V07 All other non-therapeutic products | |  |  |  |  |  |  |  |  |  |  |
| V07AB | Solvents and thinners, including rinsing solutions | 7 | 20.6 | 158 | 7.7 | 0.02 | <5 | / | 76 | 8.4 | / |

^a^ Only substances which were prescribed for at least 5 infants in cCMV_90_ are displayed.

^b^ P-value <0.05 was considered as statistically significant (Mantel–Haenszel matched-pairs test).

cCMV, congenital cytomegalovirus; cCMV_90_, infants with cCMV diagnosis during the first 90 days of life; cCMV_21-S_, infants with inpatient cCMV diagnosis and symptoms during the first 21 days of life; Controls, infants with no cCMV or CMV diagnosis in the observation period; ATC, Anatomical Therapeutic Chemical.
