# Supplementary material for: Burden of sequelae and healthcare resource utilization in the first year of life in infants born with congenital cytomegalovirus (cCMV) infection in Germany: A retrospective statutory health insurance claims database analysis
Source: PLoS One. 2023 Nov 16;18(11):e0293869. doi: 10.1371/journal.pone.0293869 (PMC10653416; doi:10.1371/journal.pone.0293869)
Supplement: S5 Table — (DOCX) [file pone.0293869.s006.docx]

S5 Table. Physical and occupational therapy prescriptions during the first 366-730 days of life.

|  | cCMV_90_ cohort | Controls | Mean  difference  (CI) | cCMV_21-S_ cohort | Controls | Mean  difference  (CI) |
| --- | --- | --- | --- | --- | --- | --- |
| No. of patients with physical and occupational therapies | | |  |  | | |
| n (%) | 5 (18.5) | 54 (4.0) |  | <5 (-) | 26 (4.3) |  |
| P-value ^a^ | <0.01 |  |  | <0.01 |  |  |
| Frequency (based on total number of patients) | | | |  | | |
| Mean | 0.7 | 0.1 | 0.6 (-0.1-1.3) | 1.2 | 0.1 | 1.1 (-0.3-2.4) |
| SD | 1.8 | 0.8 |  | 2.4 | 0.6 |  |
| Min | 0.0 | 0.0 |  | 0.0 | 0.0 |  |
| Q1 | 0.0 | 0.0 |  | 0.0 | 0.0 |  |
| Median | 0.0 | 0.0 |  | 0.0 | 0.0 |  |
| Q3 | 0.0 | 0.0 |  | 0.5 | 0.0 |  |
| Max | 7.0 | 13.0 |  | 7.0 | 10.0 |  |
| P-value ^a^ | <0.01 | |  | <0.01 | |  |

^a^ P-value <0.05 was considered as statistically significant (Wilcoxon rank-sum test for continuous variables and Mantel–Haenszel matched-pairs test for dichotomous variables).

Due to missing data, results for physical and occupational therapy prescriptions are based on n=27 infants in cCMV_90_ cohort and n=12 infants in cCMV_21-S_ cohort with valid data for physical and/or occupational therapies.

cCMV, congenital cytomegalovirus; cCMV_90_, infants with cCMV diagnosis during the first 90 days of life; cCMV_21-S_, infants with inpatient cCMV diagnosis and symptoms during the first 21 days of life; Controls, infants with no cCMV or CMV diagnosis in the observation period; CI, 95% confidence interval; SD, standard deviation; Min, minimum; Q1, 25^th^ percentile; Q3, 75^th^ percentile; Max, maximum; No, number.
