# Supplementary material for: Burden of sequelae and healthcare resource utilization in the first year of life in infants born with congenital cytomegalovirus (cCMV) infection in Germany: A retrospective statutory health insurance claims database analysis
Source: PLoS One. 2023 Nov 16;18(11):e0293869. doi: 10.1371/journal.pone.0293869 (PMC10653416; doi:10.1371/journal.pone.0293869)
Supplement: S6 Table — (DOCX) [file pone.0293869.s007.docx]

**S6 Table. Exclusion criteria for overall study population.**

| Type | Code | German Description | Group |
| --- | --- | --- | --- |
| ICD-10-GM | B20 | Infectious and parasitic diseases due to HIV disease [human immunodeficiency virus disease] | HIV |
| ICD-10-GM | B21 | Malignant neoplasms as a result of HIV disease [human immunodeficiency virus disease] | HIV |
| ICD-10-GM | B22 | Other specified diseases due to HIV disease [human immunodeficiency virus disease] | HIV |
| ICD-10-GM | B23 | Other disease states as a result of HIV disease [human immunodeficiency virus disease] | HIV |
| ICD-10-GM | B24 | Unspecified HIV disease [human immunodeficiency virus disease] | HIV |
| ICD-10-GM | Z21 | Asymptomatic HIV infection [human immunodeficiency virus infection] | HIV |
| ICD-10-GM | Z94 | Condition after organ or tissue transplantation | Transplantation |
| ICD-10-GM | T86 | Failure and rejection of transplanted organs and tissues | Transplantation |
| ICD-10-GM | U55 | Registration for organ transplantation | Transplantation |
| ICD-10-GM | Z75.6 | Registration for organ transplantation without urgency level HU (High Urgency) | Transplantation |
| ICD-10-GM | Z75.7 | Registration for organ transplantation with urgency level HU (High Urgency) | Transplantation |
| OPS | 5-335 | Lung transplant | Transplantation |
| OPS | 5-375 | Heart and heart-lung transplant | Transplantation |
| OPS | 5-504 | Liver transplant | Transplantation |
| OPS | 5-555 | Kidney transplant | Transplantation |
| OPS | 1-920.2 | Complete evaluation, with the inclusion of a patient on a waiting list for organ transplantation | Transplantation |
| OPS | 1-920.3 | Complete evaluation, with the inclusion or retention of a patient on a waiting list for organ transplantation | Transplantation |
| OPS | 5-467.6 | Small intestine transplant | Transplantation |
| OPS | 8-979 | Inpatient treatment before transplantation | Transplantation |
| OPS | 8-97c | Inpatient treatment after admission to the organ transplant waiting list | Transplantation |
| OPS | 5-525 | (Total) pancreatectomy | Other |
| ICD-10-GM | C91 | Lymphatic leukemia | Leukemia |
| ICD-10-GM | C92 | Myeloid Leukemia | Leukemia |
| ICD-10-GM | C93 | Monocytic leukemia | Leukemia |
| ICD-10-GM | C94 | Other leukemias of specified cell type | Leukemia |
| ICD-10-GM | C95 | Leukemia, unspecified | Leukemia |

ICD-10-GM, International Classification of Diseases, 10^th^ Revision, German Modification; OPS, Operation and Procedure Codes [Operationen- und Prozedurenschlüssel]; HIV, Human Immunodeficiency Virus [Humane Immundefizienz-Viruskrankheit]; HU, High Urgency.
