# Supplementary material for: Burden of sequelae and healthcare resource utilization in the first year of life in infants born with congenital cytomegalovirus (cCMV) infection in Germany: A retrospective statutory health insurance claims database analysis
Source: PLoS One. 2023 Nov 16;18(11):e0293869. doi: 10.1371/journal.pone.0293869 (PMC10653416; doi:10.1371/journal.pone.0293869)
Supplement: S7 Table — (DOCX) [file pone.0293869.s008.docx]

**S7 Table. cCMV-specific symptoms and sequelae.**

| Type | Code | Description | Sequelae |
| --- | --- | --- | --- |
| ICD-10-GM | A04 | Other bacterial intestinal infections | Intestinal disorders |
| ICD-10-GM | A09 | Infectious gastroenteritis and colitis, unspecified | Intestinal disorders |
| ICD-10-GM | D61.0 | Constitutional aplastic anemia | Anemia, neutropenia |
| ICD-10-GM | D61.3 | Idiopathic aplastic anemia | Anemia, neutropenia |
| ICD-10-GM | D61.8 | Other specified aplastic anemias and other bone marrow failure syndromes | Anemia, neutropenia |
| ICD-10-GM | D61.9 | Aplastic anemia, unspecified | Anemia, neutropenia |
| ICD-10-GM | D69 | Purpura and other hemorrhagic conditions | Purpura |
| ICD-10-GM | D70.0 | Congenital agranulocytosis | Anemia, neutropenia |
| ICD-10-GM | D70.5 | Cyclic neutropenia | Anemia, neutropenia |
| ICD-10-GM | D70.6 | Other neutropenia | Anemia, neutropenia |
| ICD-10-GM | D70.7 | Neutropenia, unspecified | Anemia, neutropenia |
| ICD-10-GM | D89 | Other disorders involving the immune mechanism, not elsewhere classified | Purpura |
| ICD-10-GM | F44.5 | Dissociative seizures | Cerebral seizures |
| ICD-10-GM | F80 | Specific developmental disorders of speech and language | Cognitive developmental disorders |
| ICD-10-GM | F81 | Specific developmental disorders of scholastic skills | Cognitive developmental disorders |
| ICD-10-GM | F82 | Specific developmental disorder of motor function | Motor development disorders |
| ICD-10-GM | F83 | Combined circumscribed developmental disorders | Motor development disorders |
| ICD-10-GM | F84 | Pervasive developmental disorders | Motor development disorders |
| ICD-10-GM | F88 | Other disorders of psychological development | Cognitive developmental disorders |
| ICD-10-GM | F89 | Unspecified disorder of psychological development | Cognitive developmental disorders |
| ICD-10-GM | G40 | Epilepsy | Cerebral seizures |
| ICD-10-GM | G41 | Status epilepticus | Cerebral seizures |
| ICD-10-GM | G80 | Infantile cerebral palsy | Paralysis |
| ICD-10-GM | G81 | Hemiplegia and hemiparesis | Paralysis |
| ICD-10-GM | G82 | Paraparesis and paraplegia, tetraparesis and tetraplegia | Paralysis |
| ICD-10-GM | G83 | Other paralytic syndromes | Paralysis |
| ICD-10-GM | H30 | Chorioretinitis | Chorioretinitis including retinal scar |
| ICD-10-GM | H31.0 | Chorioretinal scars | Chorioretinitis including retinal scar |
| ICD-10-GM | H47.2 | Optic atrophy | Optic atrophy |
| ICD-10-GM | H48.0 | Optic atrophy in diseases classified elsewhere | Optic atrophy |
| ICD-10-GM | H53 | Visual disturbances | Visual impairment |
| ICD-10-GM | H54 | Blindness and low vision | Visual impairment |
| ICD-10-GM | H90 | Conductive and sensorineural hearing loss | Sensorineural hearing loss to deafness (newborn hearing screening) |
| ICD-10-GM | H91 | Other hearing loss | Sensorineural hearing loss to deafness (newborn hearing screening) |
| ICD-10-GM | M31.1 | Thrombotic microangiopathy | Purpura |
| ICD-10-GM | P05 | Intrauterine deficiency development and fetal malnutrition | Intrauterine growth retardation |
| ICD-10-GM | P07.0 | Extremely low birth weight newborn | Intrauterine growth retardation |
| ICD-10-GM | P07.1 | Other low birth weight newborn | Intrauterine growth retardation |
| ICD-10-GM | P07.2 | Extreme immaturity of newborn (<28 week of pregnancy) | Prematurity |
| ICD-10-GM | P07.3 | Preterm [premature] newborn [other] (28-36 week of pregnancy) | Prematurity |
| ICD-10-GM | P23 | Congenital pneumonia | Pneumonia |
| ICD-10-GM | P54.5 | Skin bleeding in the newborn (ecchymoses, petechiae) | Disseminated petechiae |
| ICD-10-GM | P59 | Neonatal icterus from other and unspecified causes | Verdinikterus (direct hyperbilirubinemia) |
| ICD-10-GM | P60 | Disseminated intravascular coagulation of newborn | Purpura |
| ICD-10-GM | P61.0 | Transient thrombocytopenia in the newborn | Thrombocytopenia |
| ICD-10-GM | P61.2 | Anemia of prematurity | Anemia, neutropenia |
| ICD-10-GM | P61.4 | Other congenital anemias, not elsewhere classified | Anemia, neutropenia |
| ICD-10-GM | P61.5 | Transient neonatal neutropenia | Anemia, neutropenia |
| ICD-10-GM | P77 | Necrotizing enterocolitis of newborn | Intestinal disorders |
| ICD-10-GM | P90 | Convulsions of newborn | Cerebral seizures |
| ICD-10-GM | P91 | Other disturbances of cerebral status of newborn | Cerebral seizures |
| ICD-10-GM | Q00 | Anencephaly and similar malformations | Migration disorders of the CNS |
| ICD-10-GM | Q01 | Encephalocele | Migration disorders of the CNS |
| ICD-10-GM | Q02 | Microcephaly | Migration disorders of the CNS |
| ICD-10-GM | Q03 | Congenital hydrocephalus | Migration disorders of the CNS |
| ICD-10-GM | Q04 | Other congenital malformations of brain | Migration disorders of the CNS |
| ICD-10-GM | Q05 | Spina bifida | Migration disorders of the CNS |
| ICD-10-GM | Q06 | Other congenital malformations of spinal cord | Migration disorders of the CNS |
| ICD-10-GM | Q07 | Other congenital malformations of nervous system | Migration disorders of the CNS |
| ICD-10-GM | R16 | Hepatomegaly and splenomegaly, not elsewhere classified | Hepatosplenomegaly |
| ICD-10-GM | R23.3 | Spontaneous ecchymoses | Disseminated petechiae |
| ICD-10-GM | R56 | Convulsions, not elsewhere classified | Cerebral seizures |
| ICD-10-GM | R83 | Abnormal findings in cerebrospinal fluid | Abnormal findings in cerebrospinal fluid |

cCMV, congenital cytomegalovirus; ICD-10-GM, International Classification of Diseases, 10^th^ Revision, German Modification; CNS, central nervous system.
