# Supplementary material for: Burden of sequelae and healthcare resource utilization in the first year of life in infants born with congenital cytomegalovirus (cCMV) infection in Germany: A retrospective statutory health insurance claims database analysis
Source: PLoS One. 2023 Nov 16;18(11):e0293869. doi: 10.1371/journal.pone.0293869 (PMC10653416; doi:10.1371/journal.pone.0293869)
Supplement: S8 Table — (DOCX) [file pone.0293869.s009.docx]

**S8 Table.** **Proportions of infants with predefined sequelae during the first 1-365 days of life.**

| Sequelae | cCMV_90_ cohort | | Controls | |  | cCMV_21-S_ cohort | | Controls | |  |
| --- | --- | --- | --- | --- | --- | --- | --- | --- | --- | --- |
|  | n | % | n | % | p-value ^a^ | n | % | n | % | p-value ^a^ |
| At least one sequela | 45 | 83.3 | 1,356 | 41.9 | <0.01 | 24 | 100.0 | 633 | 44.0 | <0.01 |
| Abnormal findings in cerebrospinal fluid | 0 | 0.0 | 0 | 0.0 |  | 0 | 0.0 | 0 | 0.0 |  |
| Anemia, neutropenia | 11 | 20.4 | 53 | 1.6 | <0.01 | 9 | 37.5 | 17 | 1.2 | <0.01 |
| Cerebral seizures | <5 | / | 72 | 2.2 | / | 0 | 0.0 | 31 | 2.2 | 0.99 |
| Chorioretinitis including retinal scar | 0 | 0.0 | <5 | / | / | 0 | 0.0 | 0 | 0.0 |  |
| Cognitive developmental disorders | 12 | 22.2 | 93 | 2.9 | <0.01 | 5 | 20.8 | 55 | 3.8 | <0.01 |
| Disseminated petechiae | <5 | / | 13 | 0.4 | / | <5 | / | 7 | 0.5 | / |
| Intestinal disorders | 12 | 22.2 | 552 | 17.0 | 0.41 | <5 | / | 250 | 17.4 | / |
| Hepatosplenomegaly | <5 | / | 7 | 0.2 | / | 0 | 0.0 | <5 | / | / |
| Intrauterine growth retardation (decreased birth weight according to gestational age <3rd percentile) | 23 | 42.6 | 171 | 5.3 | <0.01 | 19 | 79.2 | 86 | 6.0 | <0.01 |
| Visual impairment | 7 | 13.0 | 74 | 2.3 | <0.01 | <5 | / | 22 | 1.5 | / |
| Migration disorders of the central nervous system | 14 | 25.9 | 43 | 1.3 | <0.01 | 10 | 41.7 | 19 | 1.3 | <0.01 |
| Motor development disorders | 18 | 33.3 | 352 | 10.9 | <0.01 | 12 | 50.0 | 158 | 11.0 | <0.01 |
| Optic atrophy | 0 | 0.0 | <5 | / | / | 0 | 0.0 | <5 | / | / |
| Paralysis | <5 | / | 21 | 0.6 | / | <5 | / | 19 | 1.3 | / |
| Pneumonia | 0 | 0.0 | 9 | 0.3 | 0.70 | 0 | 0.0 | <5 | / | / |
| Prematurity | 15 | 27.8 | 184 | 5.7 | <0.01 | 13 | 54.2 | 105 | 7.3 | <0.01 |
| Purpura | <5 | / | 18 | 0.6 | / | <5 | / | 5 | 0.3 | / |
| Sensorineural hearing loss to deafness (newborn hearing screening) | 21 | 38.9 | 72 | 2.2 | <0.01 | 9 | 37.5 | 33 | 2.3 | <0.01 |
| Thrombocytopenia | 6 | 11.1 | 10 | 0.3 | <0.01 | 6 | 25.0 | 0 | 0.0 | <0.01 |
| Verdinikterus (direct hyperbilirubinemia) | 13 | 24.1 | 377 | 11.6 | 0.01 | 9 | 37.5 | 166 | 11.5 | <0.01 |

^a^ P-value<0.05 was considered as statistically significant (Mantel–Haenszel matched-pairs test).

Infants could be diagnosed with more than one sequela.

cCMV, congenital cytomegalovirus; cCMV_90_, infants with cCMV diagnosis during the first 90 days of life; cCMV_21-S_, infants with inpatient cCMV diagnosis and symptoms during the first 21 days of life; Controls, infants with no cCMV or CMV diagnosis in the observation period.
