# Supplementary material for: Burden of sequelae and healthcare resource utilization in the first year of life in infants born with congenital cytomegalovirus (cCMV) infection in Germany: A retrospective statutory health insurance claims database analysis
Source: PLoS One. 2023 Nov 16;18(11):e0293869. doi: 10.1371/journal.pone.0293869 (PMC10653416; doi:10.1371/journal.pone.0293869)
Supplement: S9 Table — (DOCX) [file pone.0293869.s010.docx]

**S9 Table. Proportions of infants with reasons^a^ for hospitalizations during the first 1-365 days of life.**

| ICD-10-GM code | Description | cCMV_90_ cohort | | Controls | |  | cCMV_21-S_ cohort | | Controls | |  |
| --- | --- | --- | --- | --- | --- | --- | --- | --- | --- | --- | --- |
|  |  | n | % | n | % | p-value ^a^ | n | % | n | % | p-value ^a^ |
| P35 | Congenital viral diseases | 38 | 70.4 | 0 | 0.0 | N/A^c^ | 14 | 58.3 | 0 | 0.0 | N/A^c^ |
| P07 | Disorders of NB related to short gest and low birth weight, NEC | 16 | 29.6 | 152 | 4.7 | <0.01 | 14 | 58.3 | 78 | 5.4 | <0.01 |
| B25 | Cytomegaloviral disease | 11 | 20.4 | 0 | 0.0 | N/A^c^ | 9 | 37.5 | 0 | 0.0 | N/A^†^ |
| H90 | Conductive and sensorineural hearing loss | 9 | 16.7 | 23 | 0.7 | <0.01 | 6 | 25.0 | 9 | 0.6 | <0.01 |
| Z38 | Liveborn infants according to place of birth and type of del | 8 | 14.8 | 100 | 3.1 | <0.01 | <5 | / | 33 | 2.3 | / |
| P22 | Respiratory distress of NB | 6 | 11.1 | 53 | 1.6 | <0.01 | <5 | / | 29 | 2.0 | / |
| J20 | Acute bronchitis | 5 | 9.3 | 65 | 2.0 | <0.01 | <5 | / | 34 | 2.4 | / |
| H91 | Other and unspecified hearing loss | 5 | 9.3 | 13 | 0.4 | <0.01 | <5 | / | <5 | / | / |
| Z03 | Encounter for medical observation for suspected diseases and conditions ruled out | 5 | 9.3 | 20 | 0.6 | <0.01 | <5 | / | 8 | 0.6 | / |

^a^ Only inpatient primary ICD-10-GM diagnoses (3-digit), which were recorded for at least 5 infants in cCMV_90_ cohort during a hospitalization are displayed.

^b^ P-value <0.05 was considered as statistically significant (Mantel–Haenszel matched-pairs test).

^c^Comparison not applicable due to eligibility criteria.

cCMV, congenital cytomegalovirus; cCMV_90_, infants with cCMV diagnosis during the first 90 days of life; cCMV_21-S_, infants with inpatient cCMV diagnosis and symptoms during the first 21 days of life; Controls, infants with no cCMV or CMV diagnosis in the observation period; ICD-10-GM, International Classification of Diseases, 10^th^ Revision, German Modification; n, number; N/A, not applicable; NB, newborn; NEC, not elsewhere classified; w/o, without.
